# Proteasome in action: substrate degradation by the 26S proteasome

**DOI:** 10.1042/BST20200382

**Published:** 2021-03-17

**Authors:** Indrajit Sahu, Michael H. Glickman

**Affiliations:** Faculty of Biology, Technion-Israel Institute of Technology, 32000 Haifa, Israel

**Keywords:** functional characterization, molecular ultrastructure, proteasomes, ubiquitin proteasome system, ubiquitin signalling, ubiquitins

## Abstract

Ubiquitination is the major criteria for the recognition of a substrate-protein by the 26S proteasome. Additionally, a disordered segment on the substrate — either intrinsic or induced — is critical for proteasome engagement. The proteasome is geared to interact with both of these substrate features and prepare it for degradation. To facilitate substrate accessibility, resting proteasomes are characterised by a peripheral distribution of ubiquitin receptors on the 19S regulatory particle (RP) and a wide-open lateral surface on the ATPase ring. In this substrate accepting state, the internal channel through the ATPase ring is discontinuous, thereby obstructing translocation of potential substrates. The binding of the conjugated ubiquitin to the ubiquitin receptors leads to contraction of the 19S RP. Next, the ATPases engage the substrate at a disordered segment, energetically unravel the polypeptide and translocate it towards the 20S catalytic core (CP). In this substrate engaged state, Rpn11 is repositioned at the pore of the ATPase channel to remove remaining ubiquitin modifications and accelerate translocation. C-termini of five of the six ATPases insert into corresponding lysine-pockets on the 20S α-ring to complete 20S CP gate opening. In the resulting substrate processing state, the ATPase channel is fully contiguous with the translocation channel into the 20S CP, where the substrate is proteolyzed. Complete degradation of a typical ubiquitin-conjugate takes place over a few tens of seconds while hydrolysing tens of ATP molecules in the process (50 kDa/∼50 s/∼80ATP). This article reviews recent insight into biochemical and structural features that underlie substrate recognition and processing by the 26S proteasome.

## Introduction

**Figure 1.**
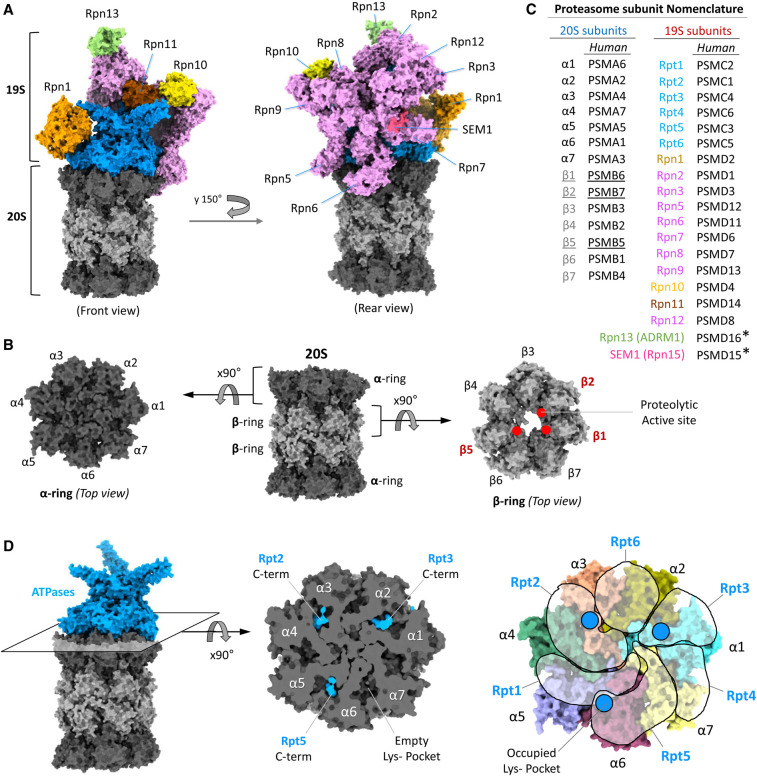
Subunit arrangement in the 26S proteasome. (**A**) The 26S is made up of a 20S catalytic core (dark and light grey) and a 19S regulatory particle (coloured). Three ubiquitin receptors (Rpn1, orange; Rpn10, yellow, and Rpn13, green) are positioned peripherally on a ‘hood’ encompassing all other Rpn subunits (Rpn11 in brown, Sem1 in rose-pink and all the rest in pink) that partially surrounds a central ATPase motor (blue). (**B**) The side view of 20S-CP shows four rings stack above other. The top view of upper α-ring (with gate closed) and upper β-ring showing the seven homologous subunits in a resting proteasome. The red dots in the β-ring show the active sites of the three catalytic subunits. (**C**) Nomenclature of 26S proteasome subunits. Subunit colour coding corresponds to that of in panel (**A**). *PSMD15 has been approved by HGNC/HUGO as an alias for SEM1. PSMD16 has been approved by HGNC/HUGO as an alias for ADRM1. (**D**) ATPase ring interaction with α-ring of 20S CP (left panel). The cross section (middle panel) at the interaction interface shows the three C-terminal tails (HbYX motif; blue) of Rpt2/3/5 inserted into the corresponding three Lys-pockets (out of seven) on α-ring. Asymmetric arrangement of six ATPases on the ring of seven α-subunits (right panel). Each transparent free-shape represents an AAA-domain of each ATPase subunit. The blue circles indicate the position of C-terminal insertion into the Lys-pocket. The figures (in panel (**A**), (**B**) and (**D**)) are generated by ChimeraX using the model structure of resting 26S proteasome (PDB: 6j2x).

Proteasomes are large protein complexes that carry out regulated intracellular proteolysis. All eukaryotic cells use proteasomes to control myriad cellular pathways by removing key proteins in a timely manner [1,2]. Proteasomes are also vital for maintaining intracellular protein quality control by removing misfolded or aggregate-prone damaged proteins [3,4]. The 26S proteasome is the major proteasome species in eukaryotes, responsible for proteolysis in the cytoplasm, in the nucleus and at the surface of most organelles [5]. Within the 26S proteasome, the 19S regulatory particle (RP) is responsible for recognising the degradation signal and unfolding of the target protein-substrate, whereas the 20S core particle (CP) hydrolyses the unfolded polypeptide into short peptides or amino acids (Figure 1A). Although the 20S CP serves as the catalytic core of the 26S proteasome, it is also quite abundant as a free 20S proteasome complex and may retain basal proteolytic activity for substrates with an unstructured or unfolded stretch [6–8]. To broaden its substrate repertoire, various activators attach to the 20S CP aiding substrate processing (SP). The 19S RP is the major activator, and either one or two 19S RPs can attach to a single 20S CP to form the singly capped 26S, or the doubly capped 30S, respectively. Nonetheless, the term 26S proteasome is generally used to refer to both 26S and 30S proteasomes. Other simpler, non-ATPase activators (e.g. PA200/Blm10, PA28/11S Reg; [9,10]) can also associate with 20S proteasomes in eukaryotes, although current understanding of their mode of action in substrate degradation is vague and will not be detailed in this review. This article focuses on recent insight into mechanisms of substrate degradation by the 26S proteasome; for a broader view of structure, function and cellular roles of 26S proteasomes, we refer readers to many recent comprehensive reviews [5,11–16].

Structurally, the 20S proteasome is a hollow cylindrical barrel consisting of four rings: two peripheral α-rings and two central β-rings made up of seven homologous subunits each (Figure 1B). In archaea and some bacteria, a simplified version of the 20S proteasome contains 14 identical copies of α and β subunits each [17,18], loosely associated with a homomeric ring of ATPases in some cases [10,19]. All 14 β subunits in archaeal proteasomes are catalytically active proteases arranged in two concerted heptameric rings around an enclosed catalytic chamber. Likewise, 14 catalytically inactive α subunits form two outer heptameric rings encompassing an antechamber. As archaea and bacteria do not encode for ubiquitin, substrate selection is independent of ubiquitin, though in some prokaryotes proteins can be converted to proteasome substrates by specific post-translational modifications [20,21]. In contrast with archaea, proteasomes in eukaryotes display greater complexity, with seven α and β paralog subunits (α1,2,3,4,5,6,7 and β1,2,3,4,5,6,7) stacked into a four-ringed 20S complex (Figure 1B). By forming an intertwined mesh at the centre of each outer α ring, the unidentical N-termini of the seven different α subunits completely seal substrate entry into the proteolytic chamber of the 20S CP [22,23]. Regarding the β subunits, only three of the seven (β1, β2, β5) retain proteolytic activity in eukaryotes (Figure 1B). Between them, they can cleave most peptide bonds since the β1 enzyme shows caspase-like (post acidic amino acid), β2 displays trypsin-like (post-basic) and β5 exhibits chymotrypsin-like (post-hydrophobic) specificities [24–27]. The formal nomenclature for α and β human counterparts is PSMA and PSMB, respectively (Figure 1C). The 19S RP is made up of a heteromeric ring of six analogous ATPases, and at least 13 extra integral subunits that stabilise the 26S holoenzyme and provide substrate specificity (Figure 1A,D) as well as a handful of transiently associated subunits [14,16,28]. Three of these integral subunits (Rpn1, Rpn10 and Rpn13) bind to the conjugated ubiquitin tag on the substrate, while three deubiquitinases (DUBs; Rpn11, USP14 and UCHL5) remove ubiquitin modifications, facilitating degradation of the conjugated substrate [14,29]. Together these features enable the eukaryotic 26S proteasome to degrade substrates in a highly regulated ubiquitin-dependent manner, ensuring its function as the major protease for intracellular proteolysis.

Structural studies have provided a wealth of information towards understating the functional complexity of proteasomes. Recent advances in Cryo-EM have revolutionized our understanding of 26S proteasomes by providing molecular maps of all 19S RP subunits, visualising conformational states based on ATPase cycles and capturing 26S proteasomes trapped with a substrate in their internal translocation channel [30–38]. In this review, we correlate recent structural information with in-depth biochemical knowledge to describe conformational changes during the functional cycle of the 26S proteasome in degrading ubiquitinated substrates.

## Signals for degradation by 26S proteasomes

Ubiquitin tagging of a protein leads to a plethora of outcomes [39,40]. A subset of ubiquitin-conjugates is targeted to the 26S proteasome for irreversible degradation in a highly specific yet regulated manner. Distinguishing between ubiquitin-conjugates is possible due to particular ubiquitin chain types that serve as preferential degradation signals. Of all linkage types, Lysine48-linked polyubiquitin is the best studied. Lysine48 linkages accumulate upon proteasome inhibition; thus, Lysine48 appears to serve as the most prevalent ubiquitin linkage for bulk degradation [41–43]. However, specific substrates or specific conditions, may entail various permutations of the ubiquitin signal. For instance, Lysine11- and Lysine29-linked polyubiquitin linkages were also found to accumulate upon proteasome inhibition and probably serve in secondary chain types for targeting to proteasomes [44–47]. Moreover, there is evidence for mixed linkages of Lysine11 or Lysine29 with Lysine48 in a single polyubiquitin chain that serve as enhanced proteasome-targeting signals [48–51]. While Lysine63-linked chains typically do not target conjugates to proteasomes in cells, these chains can associate with proteasomes and lead to proteolysis of conjugated proteins under certain conditions [52]. However, mixed Lysine63 and Lysine48 linkages in chains have been documented in the context of proteasome-dependent proteolysis [53]. Even modification by single ubiquitin units (mono-ubiquitination) has been shown to be sufficient for proteasomal degradation of select substrates [14,16,54]. Nevertheless, Lysine48-linked polyubiquitin of at least four ubiquitin units in a single chain is an efficient signal to commit typical globular proteins for degradation by the 26S proteasome and appears to be sufficient for this outcome *in vitro*, and probably also in cells [55-57].

Initial recognition and binding of the substrate is mediated primarily by the interaction of the conjugated ubiquitin chain with ubiquitin receptors on the proteasome (Rpn1, Rpn10 and Rpn13) [58-62]. Once bound, proteasome-associated deubiquitinases (pDUBs) rapidly disassemble most polyubiquitin linkages, though Lysine48-linked tetraUb stands out as it is particularly resistant, supporting its role as a robust signal for proteasome-targeting [63]. For efficient degradation, persistent ubiquitin modifications on the substrate are removed at a later stage by Rpn11, an intrinsic proteasome-resident DUB [33,38]. Although a single tetraUb chain is a sufficient signal for proteolysis, multiple ubiquitin tags on a single substrate may provide for higher binding affinity to proteasomes. In this manner, additional ubiquitin chains increase the commitment of a substrate to degradation through the simultaneous engagement of multiple ubiquitin receptors or DUBs [64,65]. Nevertheless, we note that ubiquitin is not an absolute requirement as some substrates are degraded without a ubiquitin tag, although how prevalent is ubiquitin-independent degradation in cells is still debatable. In the cellular milieu where multiple substrates compete for access to the proteasome, a hierarchy scheme is essential to provide specificity.

A secondary criterion for engagement of a substrate to the proteasome is the presence of an unstructured, unfolded or partially unfolded segment in the substrate [12,66]. Some proteins have an inherent flexible/loosely folded segment, which when compounded with ubiquitination render them efficient proteasome substrates [54,67]. Notably, even in a fully globular protein, partial unfolding may be triggered by ubiquitination [68], contributing to substrate engagement. However, the presence of an unstructured region alone without ubiquitination does not ensure high affinity of substrate binding nor efficient degradation by 26S proteasomes [54]. Hence, the ability of the 26S proteasome to recognise both a loosely folded region on the substrate and a polyubiquitin modification provides the fundamental basis for how it determines which proteins to degrade. This process is executed by two binding steps: (1) an initial, selective binding of ubiquitin chains to the receptors on the 19S RP, and (2) a subsequent secondary binding of the unstructured or loosely folded regions to the ATPases.

## Subunit arrangement in 26S proteasomes adapted for substrate degradation

As introduced above, the 26S proteasome consists of a 20S CP and a 19S cap. 30S proteasomes are complexes with a C2-symmetry, positioning 19S RP at both ends of a central 20S CP. In principle, both 19S RPs could be functional and engage with a substrate [69], however, so far SP was only shown at one side. Hence, at any given time, the 30S proteasome possibly functions as a singly capped 26S proteasome. Since the opposite side to substrate entry side most likely serves for peptide exit, concurrent functionality of both 19S RPs may be mutually exclusive. Nevertheless, this premise has not yet been adequately addressed experimentally. Therefore, in the current review, we focus only on 26S proteasome structure and function.

**Figure 2.**
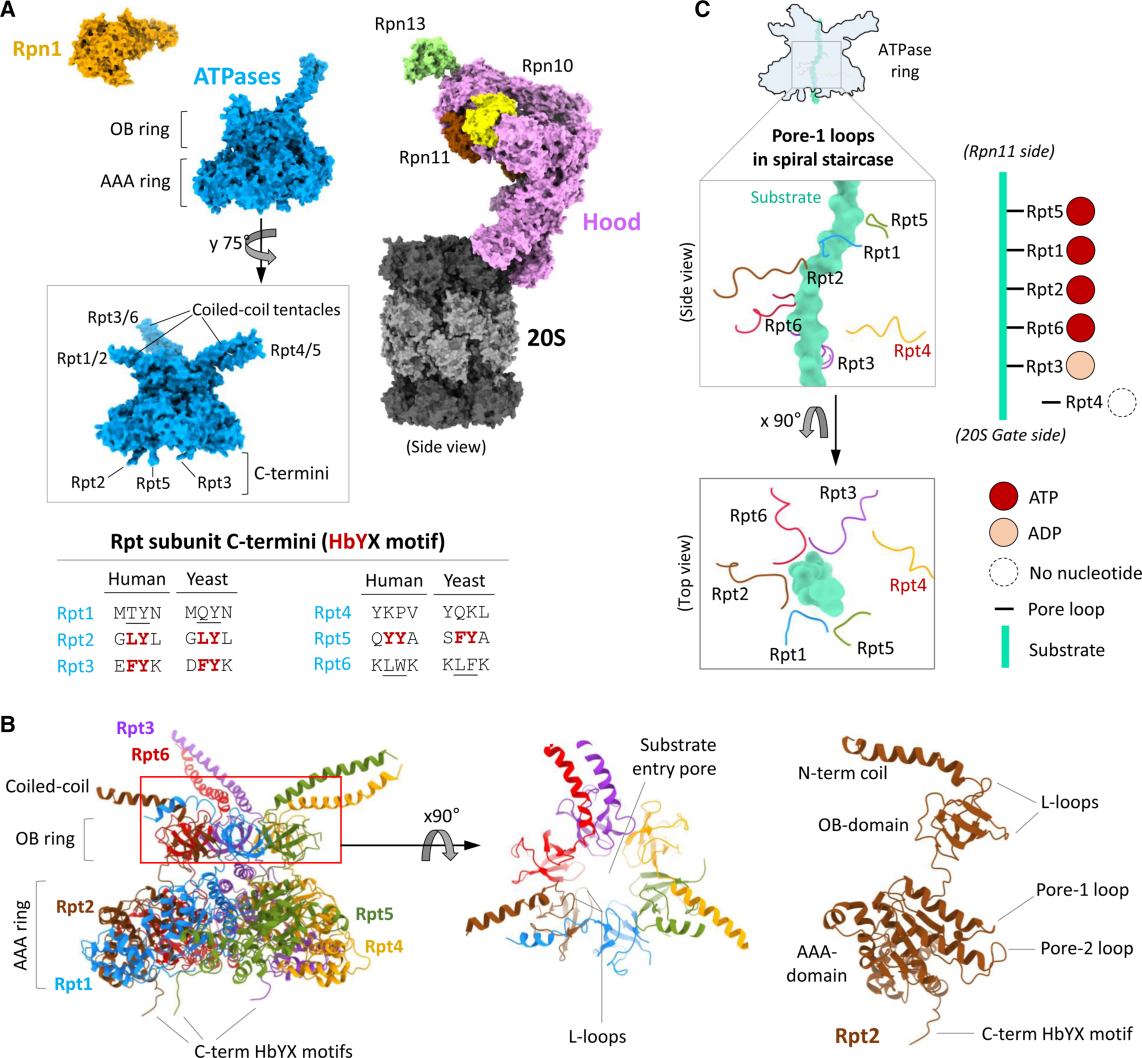
Structural details of the ATPase subunits of the 26S proteasome. (**A**) Structurally, 19S RP subunits organise into a hood (pink, brown and yellow), an engine (blue; all ATPases) and a tetraUb holder (orange; Rpn1). The insert images of ATPases ring shows the coiled-coil tentacle pairs and the three extended C-terminus. The inserted table shows C-terminal residues of Rpt2/3/5 subunits with the conserved HbYX motif in human and Yeast. The underlined C-terminal residues in Rpt1/6 are pseudo-HbYX motif. (**B**) The cartoon model of the ATPases ring shows the structural features (left). The top view of OB ring (middle) shows the central pore for substrate entry and the six pairs of L-loops. The cartoon model of Rpt2 (right) shows the detailed structural features. The figures (in panel (**A**) and (**B**)) are generated by ChimeraX using the model structure of resting 26S proteasome (PDB: 6j2x). (**C**) The model figure represents a snapshot of the pore-1 loops of all six ATPases (Rpt) arranged in a spiral staircase surrounding a substrate polypeptide (in Aquamarine colour) inside the ATPase channel during the hand-over-hand catalytic cycle. In the staircase Rpt4 pore-1 loop is disengaged from the substrate. The figure is generated by ChimeraX using the model structure of substrate bound 26S proteasome (PDB: 6msk).

The 19 RP is made up of 19 integral subunits including six proteasome AAA-ATPases (Regulatory Particle Triple-A ATPases; Rpt1-6) and 13 non-ATPase subunits (Regulatory Particle Non-ATPases; Rpns) (Figure 1A). The formal nomenclature for their human counterparts is PSMC and PSMD, respectively (Figure 1C). The six homologous ATPases are arranged in a hexameric ring placed over the α-ring of the 20S CP, covering the entrance through which substrates enter (Figure 1D). Due to the symmetry mismatch between six ATPases and seven α subunits, the ATPase ring is slightly offset in the resting state of the proteasome, obstructing substrate traffic through the translocation channel into the 20S proteolytic chamber. Structurally the ATPase ring itself consists of an upper OB ring and a lower AAA ring (Figure 2A), which are misaligned between themselves in the resting state. The N-terminus of each ATPase extends beyond the OB ring as a long (∼60 aa) α-helix, paired with its immediate neighbour (Rpt1/2, Rpt3/6 and Rpt4/5) to form coiled-coil structures. These three coiled-coil tentacles play vital roles in interacting with other Rpn subunits. Specifically, the Rpt1/2 coiled-coil interacts with Rpn1 while the Rpt3/6 coiled-coil interacts with Rpn2. The Rpt4/5 coiled-coil does not interact in the resting state with other subunits, therefore, is free to provide contact for substrates or possibly with the ubiquitin tag [70]. The three coiled-coils meet at their base to form the aperture of the OB ring, the port of substrate entry [32]. The pore of the OB ring lined with six pairs of L-loops (Figure 2B) which usually blocks the entry of folded proteins but suggestively initiate entry of an unfolded/unstructured segment of the substrate by establishing interactions [31]. In the lower AAA ring each Rpt subunit contains two vertically arranged substrate interacting loops called pore-1 loop (upper position) and pore-2 loop (lower position) [31]. The six pairs of pore loops are arranged in two anticlockwise spiral staircases (Figure 2C) [71]. The staircase arrangement enables the unidirectional translocation of the substrate polypeptide through the ATPase channel. The ATPase ring is pegged onto the surface of the 20S CP through HbYX motifs at the extreme C-termini of three of the six ATPases (Rpt2, Rpt3 and Rpt5; Figure 2A) that insert into corresponding lysine-pockets [16]. Specifically, Rpt2 HbYX inserts into the lysine pocket at the interface of α3/4, Rpt3 into α1/2 and Rpt5 into α5/6 [72-74] (Figure 1D). During the ATPase cycle additional insertion of the extreme C-termini (pseudo HbYX motif; Figure 2A) of Rpt6 into the lysine pocket at the interface of α2/3 and of Rpt1 into the lysine pocket at α4/5 completes gate opening and facilitates substrate translocation [32,36,75].

All Rpn subunits — except for Rpn1 — form a hood-like structure, positioned at one side of the proteasome extending from the 20S α-ring over the ATPases ring. Rpn1 attaches to the ATPase ring opposite to this hood, providing multiple binding sites: for ubiquitin modifications [60,61], the tetraUb signal [35], Ubl-containing proteins [60,62,76] and the deubiquitinase USP14/Ubp6 [31,60,77]. A typical 26S proteasome contains three ubiquitin receptors — Rpn1, Rpn10 and Rpn13. Rpn10 (UIM domain) and Rpn13 (Pru domain) provide monoUb- or diUb-binding sites [16,78], whereas Rpn1 provides multiple ubiquitin-binding sites: both monoUb and diUb at a T2 motif [60,61], and tetraUb at the PC crown [35]. Some reports show that Rpn10 or Rpn13 subunits are labile, attaching and detaching such that in some instances they may be substoichiometric, implying that not all 26S holo-complexes contain either or both of these subunits [79,80]. Counteracting the ubiquitin signal, the 19S RP also harbours a few DUBs [14,29]. Rpn11 is an integral DUB [81] positioned adjacent to the substrate entry port of the ATPase OB ring [33]. Typically, Rpn11 efficiently removes the entire ubiquitin chain by en-bloc removal of the last remaining ubiquitin chain or residue from the translocating conjugate to facilitate entry of the substrate and recycling of the ubiquitin molecule [33,81-83]. Interestingly, a fixed ubiquitin chain that was resistant to Rpn11 cleavage did not block substrate entry or degradation, but rather resulted in the unfolding of the conjugated Lysine48-linked ubiquitin chain and its degradation along with the substrate [56].

**Figure 3.**
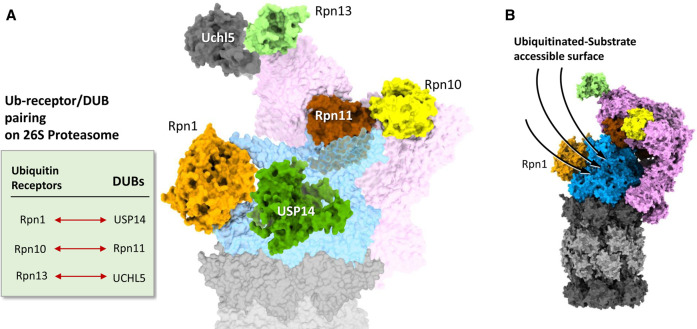
Features of 26S proteasomes adapted for substrate binding. (**A**) Ubiquitin-receptor and deubiquitinase pairing on 26S proteasome. Ubiquitin receptors and deubiquitinases are positioned in pairs (Rpn1-USP14, Rpn10-Rpn11 and Rpn13-Uchl5). Models of 26S proteasome are by ChimeraX from PDB: 6j2x, Usp14 from 5gjq (Ubl-domain missing), and Uchl5 from 3ihr. Usp14 and Uchl5 models are simply placed over the proteasome model according to their corresponding positions. (**B**) A side view of a 26S proteasome showing the putative surface accessible for ubiquitinated substrate binding. Model of 26S proteasome is generated by ChimeraX using the PDB: 6j2x.

Apart from the integral DUB — Rpn11, there are other pDUBs — USP14 and UCHL5, which bind transiently to the 19S RP during the degradation process [84,85]. USP14/Ubp6 is likely targeted to the Rpn1 subunit via an N-terminal UBL domain similar to many other UBL-containing proteins that transiently associate with proteasomes [86]. However, in Cryo-EM images of USP14-containing 26S proteasomes, this UBL domain was not visualised (Figure 3A), suggesting that it is either flexible or takes-up multiple conformations [31,77]. Moreover, the UBL domain is not strictly essential for binding to proteasomes as the USP domain maintains interactions with the OB ring of RPT subunits, in particular with Rpt1 [31,77], and indeed, truncated USP14 lacking its N-terminal UBL can associate with proteasomes [87,88]. USP14 is not constitutively active but is allosterically activated when incorporated into the 26S proteasome [77]. On the proteasome, USP14 removes ubiquitin chains from substrates modified by multiple chains, until a last chain remains [64]. A single modification of four ubiquitin units or more linked via lysine-48 is relatively resistant to USP14 activity [63], suggesting that the last remaining chain would generally be K48-linked tetraUb. UCHL5 (Uch37) is activated upon binding to the proteasome where it interacts with Rpn13 [89-91]. UCHL5 primarily deubiquitinates K48-linked ubiquitin chains [92], and exclusively branched chains [93]. In this manner, both USP14 and UCHL5 are thought to act on a bound substrate removing superfluous or auxiliary ubiquitin modifications to decrease the possibility of substrate stalling during translocation into the ATPase pore.

**Figure 4.**
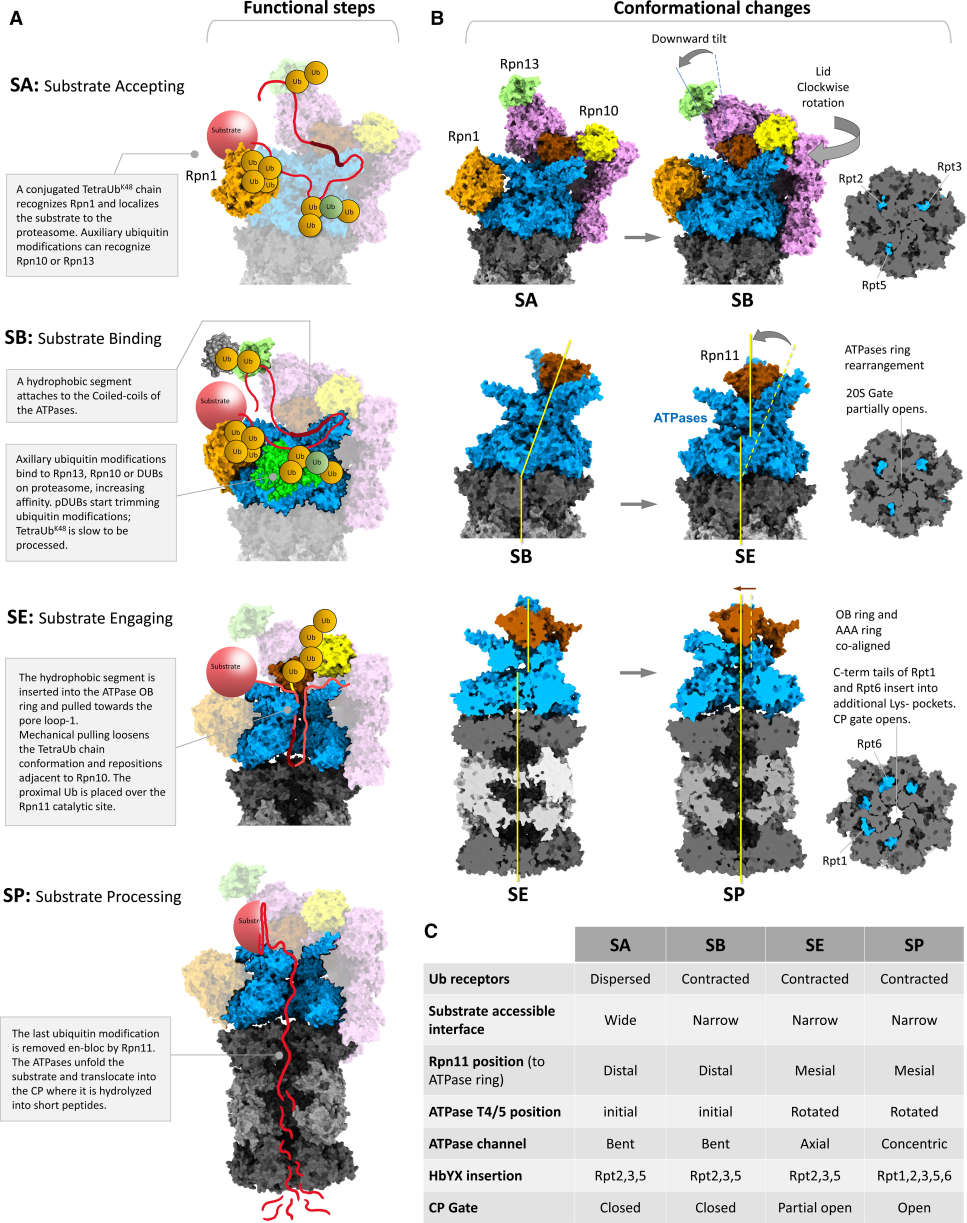
Functional steps and conformational changes during substrate processing by 26S proteasomes. (**A**) We describe four steps during proteasome function: SA, substrate accepting; SB, substrate binding; SE, substrate engagement and SP, substrate processing. The substrate model illustratively depicts a multiple ubiquitinated-conjugate modified by a Lysine48-linked tetraUb and a branched-mixed polyubiquitin chain. (**B**) Illustration of conformational changes during transition from one step to the next. Proteasome models are generated using different PDB structures of 26S proteasomes in various conformations (PDB: 6j2x, 6j30 and 6j2n). The three cross-sectional views of 20S CP near upper α-ring show; a three-tail insertion with close gate (SA), a three tail-insertion with partial open gate (SE) and a five-tail insertion with open gate (SP) are generated using PDB: 6j2x, 6j30 and 6msk, respectively. (**C**) A table summarising the major characteristic features of each functional step following substrate binding.

**Figure 5.**
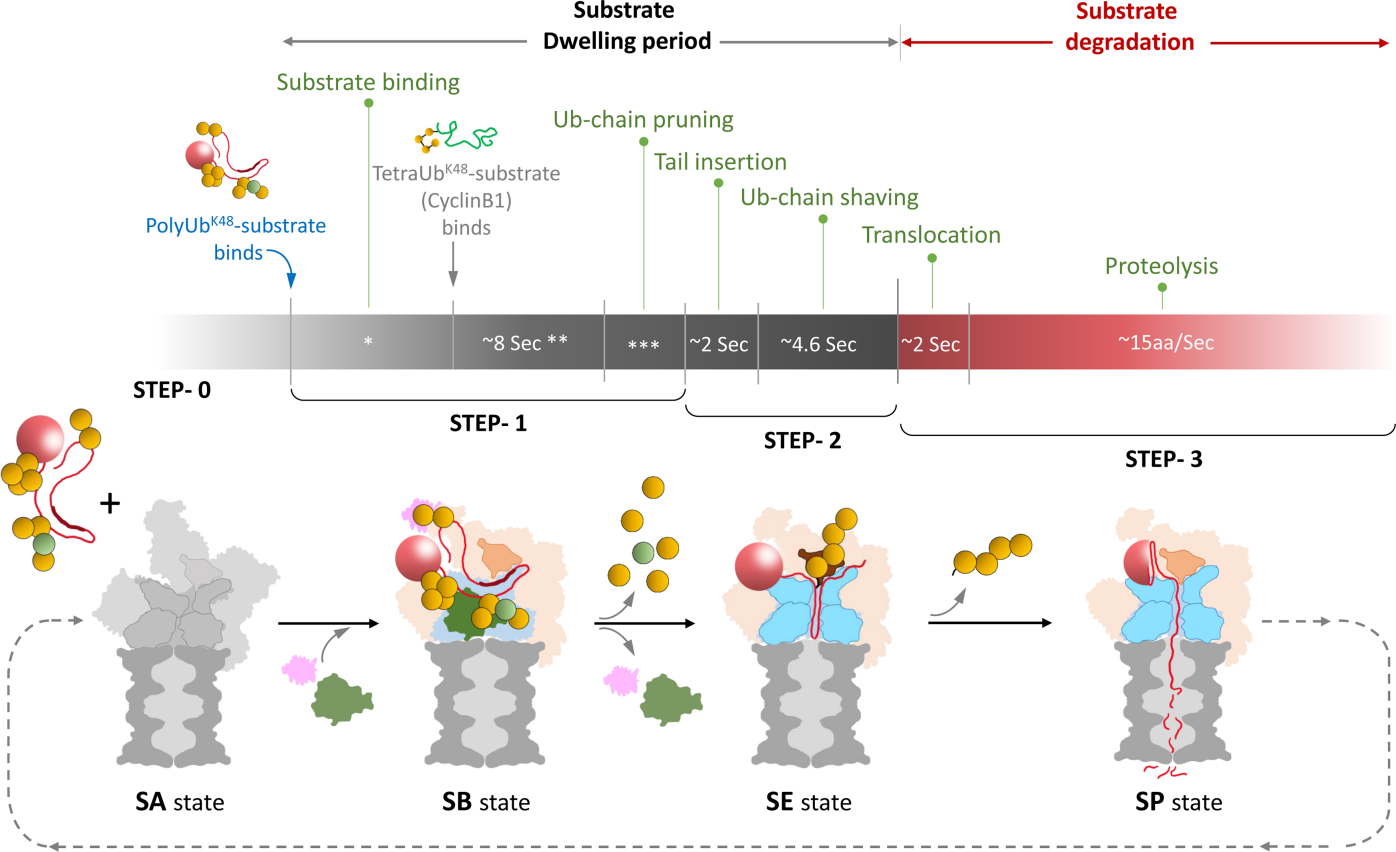
Substrate degradation kinetics of a 26S proteasome. The full functional cycle can be divided into two phases — (1) Substrate dwelling period (2) substrate degradation period. Substrate dwelling period comprises the Step0, step1, step2 of substrate processing with SA, SB and SE conformational changes to the 26S proteasome. Proteasome-associated DUBs (pDUBs) incorporate along with the substrate. In this period multiple events take place such as substrate recognition, binding, Ub-chain pruning, tail insertion and Ub-chain shaving (see Figure 2). During the substrate degradation period (Step3: SP-state) substrate translocation and proteolysis continues until complete degradation. After one functional cycle, the proteasome likely returns to the resting state/SA-state for the next cycle. For a typical 50 kDa protein attached to a single Lysine48-linked tetraUb chain, the entire process takes ∼50 s and uses up to ∼80 molecules of ATP. The symbols * and *** represent variable time frames depending on the nature of polyubiquitination. The symbol ** represents the time (8 s) requirement for binding of a typical substrate with a single Lysine48-linked tetraUb chain.

It has been reported that incorporation of USP14 (even catalytically inactive USP14) allosterically suppresses proteasome ATPase activity [94]. After binding of a ubiquitin conjugate to the 19S RP, association of a ubiquitin unit to the active site of USP14 leads to ATPase stimulation [95,96] triggering an ATP-dependent transition to the substrate engaged state. Other studies showed that the UBL domain of USP14 alone, is sufficient to stimulate ATPase activity and activate proteolysis by 26S proteasomes [86,97]. Regardless of whether triggered by the UBL or the activated USP domain, these allosteric effects enhance 26S proteasome specificity for ubiquitinated proteins over non-specific proteolysis of unmodified substrates.

The need to disassemble diverse ubiquitin linkages on substrates leaves open the possibility that other DUBs play auxiliary roles in proteasome function too. Interestingly, when incorporated into the proteasome, all three pDUBs pair with one of the three ubiquitin receptors: Rpn11 with Rpn10, USP14 with Rpn1 and Uchl5 with Rpn13 (Figure 3A). The pairing of DUBs with Ub-receptors synchronises the removal of ubiquitin for optimal degradation of the target without degrading the ubiquitin signal. Overall, the proteasome subunit arrangement provides multiple attachment sites for recognition and engagement of diverse substrate elements, leading to the guided entry of substrate to the internal translocation channel (Figure 3B).

## The functional cycle of 26S proteasomes

Single-particle Cryo-EM analysis of proteasomes bound to ubiquitin, substrates or nucleotides, combined with biochemical information about proteasome function, define the catalytic cycle with the following functional states: substrate accepting (SA), substrate binding (SB), substrate engaging (SE) and substrate processing (SP) states. We classify the above states based on the functional characteristics of 26S proteasome while handling a substrate (hence we do not describe all the reported conformational states of 26S proteasome based on various structural approaches). Undoubtedly, additional sub-classifications will emerge as new information is put forth.

### Substrate accepting (SA) state

At any given cellular condition, not all proteasomes are at work. For instance, Baumeister's group has suggested that in neuronal cells roughly 60% proteasomes are in a resting state [69] (although this number may vary in actively dividing cells). We also note that ‘resting proteasome’ is functionally the ‘substrate accepting’, and may in fact encompass a range of sub-conformations [30,34,37,98,99]. These resting proteasomes are ready to accept substrate and enter into a new functional cycle (Figure 4A). The SA state is characterised by a peripheral distribution of the three ubiquitin receptors on the 19S RP, and a wide-open surface at one side of the ATPase ring that is accessible to the incoming substrate (Figure 4B,C). What may be the benefit of dispersing the ubiquitin receptors peripherally on the 19S RP? Such an arrangement likely increases the probability of recruitment, and once bound, provides anchors for multiple ubiquitin chains attached to a single substrate. Although single particle Cryo-EM studies of 26S proteasomes in this state reached a resolution of 3.5 Å [35,99], Rpn1 and Rpn13 have been found at lower resolution than the rest of the 19S RP. The apparent dynamic behaviour of these two ubiquitin receptors is a hallmark of the SA state, ostensibly to provide the flexibility needed for binding of heterogeneous types of ubiquitin chains or linkages. Moreover, in this conformation, Rpn11 positions distal to the ATPase pore, the ATPase channel is bent and misaligned to the closed 20S CP gate (Figure 4B). Therefore, while the SA state may be ideal for receiving ubiquitin-conjugates, it is less-than-optimal for SP and translocation. Subsequent conformational changes alleviate this ‘auto-inhibition’.

### Substrate-binding (SB) state

After encountering a ubiquitinated substrate, the 26S proteasome enters into a SB state. With multiple ubiquitin-binding sites, and an inherent affinity for Lysine48-linked tetraUb, Rpn1 emerges as the most likely port for SB. In support of this hypothesis, Baumeister's group reported extra electron density at Rpn1 attributed to a substrate (Poly-GA aggregate) bound to 26S proteasomes in neuronal cells [69]. Lysine48-linked polyubiquitin binding alone was sufficient to trigger a series of conformational changes at the 19S RP that are interpreted as competent to trap the conjugated substrate on the proteasome [32,35,38]. This movement brings the three ubiquitin receptors closer together, increasing affinity for the substrate by providing multiple attachment sites to the ubiquitin units on the substrate (Figure 4B). The resulting SB state is characterised by a stable electron density of the Rpn1 subunit, a tilted movement of Rpn2 and Rpn13 towards Rpn1, and a twisted movement of the backside ‘Lid’ subunits narrowing the exposed ATPase surface, while the ATPase ring remains in a bent conformation [35]. Notably, studies employing Lysine63-linked substrates did not describe these early events upon SB [32,38].

Proximity pairing of each ubiquitin receptor with a DUB enhances affinity for ubiquitin-conjugates while facilitating disassembly of ubiquitin chains. In addition to the recycling of conjugated ubiquitin, deubiquitination removes potential obstructions for subsequent substrate translocation. Between them, these pDUBs display a broad specificity for a variety of linkages and lengths, with a notable exception of Lysine48-linked tetraUb that is slow to be processed [63]. This property of pDUBs guarantees a longer residency time of Lysine48-linked tetraUb conjugates, to prepare the target substrate for unfolding and degradation. While a Lysine48-linked tetraUb effectively provides sufficient dwelling time for the ATPases to engage the substrate at a loosely folded stretch, auxiliary ubiquitin modifications on the substrate are removed by the pDUBs [64].

### Substrate engaging (SE) state

After initial recognition and successful binding of a substrate to the 26S proteasome, an unstructured stretch of the substrate engages to the entry pore of the ATPase ring (Figure 4A). This stretch from either termini or from a mid-loop [100], encounters the OB ring L-loops lining the entry port into the ATPase channel [31,101]. Subsequent ATP-driven pulling forces by pore-1 and pore-2 loops pull the polypeptide deeper into the ATPase channel until the proximal ubiquitin unit of the last remaining ubiquitin chain attached to the substrate is positioned near Rpn10 [32,38]. Ubiquitin binding to Rpn10 may be enhanced by ‘opening’ [102,103] of the ‘closed tetraUb conformation’ [104,105] to expose the hydrophobic patches of individual ubiquitin units. At this stage, the substrate is fully engaged and committed for destruction. The importance of Rpn10 to the processing of substrate was elegantly reported by Matouschek and co-workers [62]. Once the proximal ubiquitin encounters the ATPase pore, a translocation coupling mechanism repositions Rpn11 to access the isopeptide bond between the ubiquitin modification and the substrate [33,82]. As a result, the last ubiquitin chain (usually a tetraUb) is shaved en-bloc from the substrate. In most cases, ubiquitin is rescued from degradation by this mechanism, but if escapes Rpn11 cleavage, it may be unravelled and degraded along with the substrate [56].

Mechanistic insight for substrate translocation was recently reported by Martin's group and Mao's group identifying various substrate-bound conformational sub-states where each ATPase subunit was in a different ATP hydrolysis or substrate-interacting conformation [32,38]. This resolution was made possible either by chemically inhibiting Rpn11, or by stalling ATPase hydrolysis with ATPγS. The resolved conformational sub-states reflect dynamic movements of the ATPase that serve to unfold the substrate as well as trans-activate Rpn11 for removal of the remaining proximal ubiquitin unit that hinders substrate translocation into the 20S gate. In a resting/SA state, all six proteasomal ATPases are nucleotide-bound conformation: 5 ATPases with ATP and one ATPase with ADP [36]. The ADP-bound Rpt pore-1 loop is usually positioned at the bottom of the pore-loop staircase. Post engagement at the OB ring, the substrate interacts with the pore loops of the AAA ring. During an ATPase-cycle the second-lowest positioned pore-1 loop containing Rpt (for example Rpt3, Figure 2C) hydrolyses the bound ATP molecule to ADP and shifts the pore-1 loop to the lowest position in the staircase, while the lowest positioned pore-1 loop containing Rpt (for example Rpt4) releases ADP and is disengaged from the substrate polypeptide. Subsequent rebinding of ATP to the disengaged Rpt repositions the pore-1 loop to the top of the staircase where it is re-engaged to the substrate. This hand over hand movement continues anticlockwise (for example, from lowest to highest Rpt4–Rpt3–Rpt6–Rpt2–Rpt1–Rpt5), and with each ATP hydrolysis and ADP release the polypeptide is mechanically pulled towards the 20S CP gate. As mentioned above, engagement of substrate to the OB ring correlates with greater conformational changes characterised by co-alignment of OB ring with the AAA ring, repositioning of Rpn11 on the ATPase pore, movement of pore-loops and partial opening of the 20S CP gate (Figure 4B,C).

### Substrate processing (SP) state

Substrate-engaged proteasome elicits insertion of Rpt1 and Rpt6 C-termini into the corresponding lysine-pockets of the 20S CP α-ring (in addition to the already inserted Rpt2, Rpt3 and Rpt5 C-termini), completing 20S gate-opening [32,36,75]. In this state, the ATPase channel is fully contiguous with the translocation channel of the 20S CP (Figure 4B). The pulling force by the six proteasomal ATPases continues to unfold the substrate and translocate through the 20S CP cis α-ring. To reach the β-catalytic chamber, the substrate traverses through the opened α-annulus, the antechamber and the β-annulus. During this journey, the substrate most likely encounters other residues protruding from the inner wall of the 20S CP. Within the catalytic chamber, the proteolytic β active-sites (β1, β2 and β5) cleave the substrate polypeptide into short peptides ranging from 5 aa to 35 aa long [106,107]. Although shorter peptides or even free amino acids are probably generated, current mass spectrometry approaches have not yet addressed the identity of peptides shorter than 5 aa long. Peptide products have been proposed to exit from the other end of the 20S CP (through trans-α-ring), although definitive evidence has yet to be put forth. Current Cryo-EM analysis has not identified conformational changes or electron density loss at the side walls of the 20S CP during SP that could support the alternative hypothesis that peptide products diffuse out through the side walls. Hence, for the symmetric doubly capped 30S proteasome to function efficiently, the substrate should enter from one side as peptide exits from the other. Although proteasome particles were identified with substrates engaged at both 19S RPs, as demonstrated in neurons with poly-GA aggregates attached to both sides of 30S proteasomes [69], these may represent stalled proteasomes leaving the issue still unresolved whether both sides of 30S proteasome are simultaneously productive for SP.

After deubiquitination, unfolding, translocation and proteolysis of an entire substrate, a proteasome completes one degradation cycle and is set for another catalytic cycle. In the transition phase, the 26S proteasome gradually regains the initial resting conformation although the time taken for this reversal is unclear. The setback process could be either in a stepwise manner (SP–SE–SB–SA), or directly from SP to SA, but awaits further investigation.

## Substrate degradation kinetics by 26S proteasomes

The entire degradation/functional cycle detailed above, is an energy-consuming process. To execute each step, 26S proteasomes acquire necessary conformational changes initiated by SB and powered by ATP hydrolysis. Given the complexity of each protein-substrate, every degradation cycle may take a different duration to complete. The major factors affecting the rate of proteolysis of a single substrate molecule are: type of ubiquitin linkages (length, homogenous, mixed or branched), size of the protein-substrate, folding or presence of unstructured regions. A few studies have meticulously addressed the kinetics of 26S proteasomes degrading a model substrate [65,108,109]. Here, we comprehensively integrate the current understanding of 26S proteasome kinetics for a typical substrate with an unstructured stretch and modified by Lysine48-linked polyubiquitin chains with at least one tetraUb chain (Figure 5). The proteasome considered herein is a singly capped 26S and with its typical associated DUBs.

The moment a polyubiquitinated substrate encounters the 26S proteasome, the countdown for its destruction begins. Usually, the total time spend by a typical substrate on a proteasome can be divided into two phases — (1) substrate dwelling phase and, (2) substrate degradation phase. Variables that affect substrate dwelling time are the types of ubiquitination and the nature of the unstructured stretch. A substrate (CyclinB1) conjugated with a typical Lysine48-linked tetraUb chain, spends <10 s before deubiquitinated by Rpn11 [65]. The presence of additional ubiquitin chains would add dwelling time providing higher affinity for proteasomes [64]. In the cellular milieu, many of these additional chains probably include mix/branched linkages increasing the efficiency of commitment [49,50,110]. These axillary polyubiquitin chains are pruned by the pDUBs during substrate engagement (for ubiquitin recycling and ease of substrate translocation) which add to the total dwelling time. A sufficient dwelling time enables the conformational changes on proteasomes to facilitate the insertion of an unstructured segment into the ATPase channel, which has been estimated to take ∼2 s [108]. Within this timeframe, the polypeptide tail enters through the OB ring and engages the ATPase pore loops. Post engagement, the polypeptide continues to translocate through the ATPase channel at a rate of 15 aa/s until the proximal isopeptide bond of the conjugated ubiquitin chain encounters Rpn11 at the ATPase pore [108]. Hence, this translocation duration depends on the distance from the inserted tail to the position of conjugated ubiquitin. At this stage, translocation is hindered unless the last polyubiquitin chain is shaved ‘en-bloc’ by Rpn11 [33,64,82], estimated at ∼5 seconds based on an anisotropy FRET assay [16]. Even in the absence of associated pDUBs on proteasomes, Rpn11 is efficient enough to remove multiple ubiquitin chains from the substrate in discrete steps [65] but at the expense of extra time for proteolysis. Inhibiting Rpn11 DUB activity has been used to trap proteasomes with an actively translocating substrate [38]. Nevertheless, this DUB activity is not a prerequisite for processivity since a substrate with a DUB-resistant ubiquitin modification was processed to completion [56]. As a consequence, multiple polypeptides attached at a branch-point would need to translocate simultaneously through the gated channel thereby slowing down the overall translocation rate.

The kinetics of the substrate proteolysis phase are simple and straight forward. Once all the ubiquitin chains are removed from the substrate, the six ATPases unfold and translocate it continuously at a rate of ∼15 aa/s motorised by ATP hydrolysis. In general, the rate of peptide bond hydrolysis by the 20S CP active sites is proportional to the rate of ATP hydrolysis by the 19S RP [109]. For a typical substrate, in each ATPase cycle two ATP molecules are hydrolysed by two of the six ATPases (Rpts) in 0.8 s, driving translocation of 12 aa residues into the proteolytic chamber. However, the rate of ATP hydrolysis may vary from substrate to substrate depending on the globularity of the substrate. For example, at Vmax, a purified 26S proteasome takes ∼50–80 ATP molecules and ∼23 s to degrade a ubiquitylated DHFR molecule. If, DHFR binds to its substrate folate and thus assumes a more tightly folded conformation, the number of ATP molecules consumed and the time required to degrade DHFR doubles [109]. The greater ATP consumption with the more tightly folded substrate occurs not because the proteasome hydrolyses ATP at a higher rate but because the overall degradation process takes longer.

## Perspectives

- *Importance of the field*: Upon completion of their tasks, most cellular proteins are removed in a ubiquitin-dependent manner by the 26S proteasome, making it one of the most powerful regulators of cellular activity.
- *Current thinking*: The primary signal that targets substrates for degradation by 26S proteasome is ubiquitin modification. Upon binding of a polyubiquitin conjugate, the proteasome enters a series of conformational changes to recycle the ubiquitin tag and to engage the substrate irreversibly for its destruction.
- *Future directions*: Further dissection of the 26S proteasome mechanism awaits single-particle analysis of 26S proteasomes combined with a set of well-characterized ubiquitinated substrates of defined properties to unravel proteasome-in-action.

## Footnote

PSMD15 has been approved by HGNC/HUGO as an alias for SEM1. PSMD16 has been approved by HGNC/HUGO as an alias for ADRM1.

## Abbreviations

DUBs: deubiquitinases
pDUBs: proteasome-associated deubiquitinases
RP: regulatory particle
SA: substrate accepting
SB: substrate binding
SE: substrate engaging
SP: substrate processing
